# Utilization of single-use gowns reduces the incidence of postoperative infections

**DOI:** 10.1186/1749-8090-8-S1-P79

**Published:** 2013-09-11

**Authors:** M Nedić, H Gašparović, L Svetina, K Čuljak, R Habeković

**Affiliations:** 1 The University of Zagreb, School of Medicine, Zagreb, Croatia; 2 Department of Cardiac Surgery University Hospital Center Zagreb, Zagreb, Croatia

## Background

Gowns and drapes are used widely in health care facilities. Gowns have been used to minimize the risk of disease acquisition by health care providers, to reduce the risk of patient-to-patient transmission, and to maintain sterility during invasive procedures. The aim of this prospective randomized study is to compare the ability of single-use and reusable surgical gowns to protect against the infections during surgical procedures in the Department of Cardiac Surgery University Hospital Center Zagreb.

## Methods

We conducted a prospective observational study. Group 1 consisted of patients protected with single-use surgical gowns during surgical procedures. Group 2 consisted of patients protected with reusable surgical gowns during surgical procedures. Information obtained preoperatively included demographic data, surgery related data and postoperative complication occurrence and outcome data. During recovery, the incidence of postoperative infections was observed. Six weeks postop, patients were examined for the occurrence of urinary tract infection, central vein catheter and wound infections, pneumonia, sepsis, and other non-specific infections.

## Results

Correlation between groups and infection occurrence in a 6-week postpone period was analyzed. Odds ratio was used; OR 0.3214, CI (95%) = 0.1273-0.8113, p = 0.0163. Use of single-use gowns and drapes reduced the incidence of the primary adverse outcome. Among patients who developed infections, 58.82% of underwent coronary artery bypass graft surgery, 35.29% underwent valve related procedures, and 5.88% underwent other types of surgery.

## Conclusion

The occurrence of infections is 67.86% lesser in group with single-use surgical gowns. More liberal use of single-use gowns should be implemented in contemporary protocols designed to reduce the incidence of postoperative infections.

